# Visceral leishmaniasis elimination in South Asia: lessons learnt can inform disease elimination in East Africa

**DOI:** 10.1136/bmjgh-2026-023521

**Published:** 2026-03-19

**Authors:** Piero Olliaro, Dinesh Mondal, Ermias Diro, Winnie Mpanju-Shumbusho

**Affiliations:** 1 Nuffield Department of Medicine, University of Oxford Pandemic Sciences Institute, Oxford, UK; 2 Parasitology Laboratory, International Centre for Diarrhoeal Disease Research, Bangladesh, Dhaka, Bangladesh; 3 Department of General Internal Medicine, University of Washington—Seattle Campus, Seattle, Washington, USA; 4 Uniting to Combat Neglected Tropical Diseases, London, UK

**Keywords:** Public Health

## Abstract

In 2005, the governments of Bangladesh, India and Nepal, in partnership with the WHO, started the Kala-azar Elimination Programme (KEP) to reduce the incidence of visceral leishmaniasis to below 1 new case in 10 000 population. The target was achieved by Bangladesh in 2017 and validated in 2023. The KEP has demonstrated that, through a concerted approach and public–private partnership, it is possible to eliminate visceral leishmaniasis from the world’s highest endemic region, Southeast Asia. The experience learnt can be used elsewhere for visceral leishmaniasis as well as for other diseases targeted for elimination.

**Summary box**

To be successful, a disease elimination programme requires the following.

- Assessing the available interventions and setting realistic expectations for what they can and cannot achieve thus requiring investments in to new tools to be developed.
- Ensuring the sustainability, scalability and affordability of the interventions.
- Guaranteeing unwavering political commitment and proportionate, coordinated, sustained funding and cross-border collaboration.
- Realising that major public health achievements cannot happen without the concerned countries playing a central role in the planning and execution of public health interventions.

## Introduction

 Visceral leishmaniasis (VL, also known as kala-azar), if untreated, is a debilitating and potentially lethal parasitic disease. Since the earliest documented outbreaks in India in the first half of the 19th century, South Asia became the region with the highest VL burden in the world, contributing about 60% of the total estimated 300 000–500 000 annual cases until the beginning of the 21st century.^1^ In 2005, the governments of Bangladesh, India and Nepal, in partnership with the WHO and its Special Programme for Research and Training in Tropical Diseases (TDR), committed to eliminating VL as a public health problem by 2015. The resulting Kala-azar Elimination Programme (KEP) had the target to reduce VL incidence below 1 new case in 10 000 population at different administrative levels in each country.^2^

**Figure 1:**
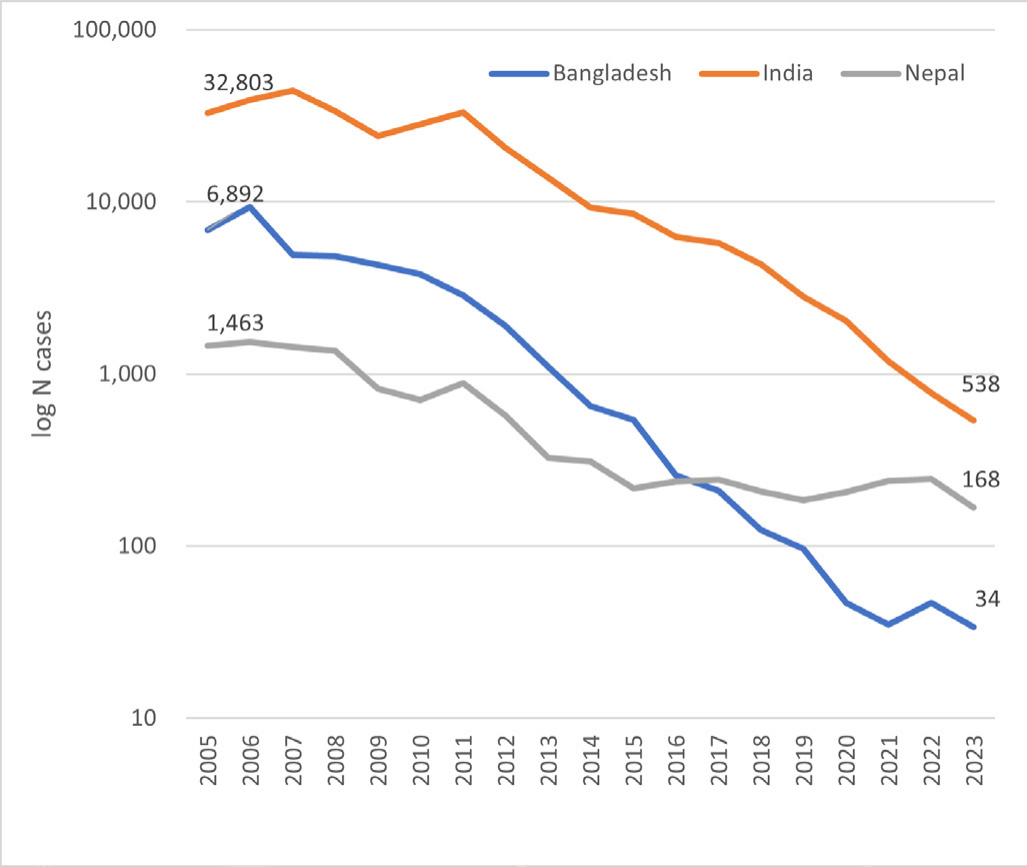
Number of reported visceral leishmaniasis cases in Bangladesh, India and Nepal, 2005–2023. Source: WHO Global Health Observatory https://www.who.int/data/gho/data/themes/topics/ghontd-leishmaniasis.

In 2017, Bangladesh became the first country ever to reach the target, and sustained elimination was validated by WHO in 2023.^3^ Concomitant efforts in India and Nepal have radically changed the burden in those countries and worldwide.^4^ Compared with before these efforts, by 2023, Bangladesh had reduced the number of cases by 99.5%, India by 98.4% and Nepal by 88.5%; regionwide, there remains a mere 2% of pre-KEP levels (figure 1). This success has now shifted the global burden of VL to the East African countries, which is the next target region for VL elimination.^5^

Inclusive internationalism is a collaborative approach that ensures fair representation and active participation of all countries in global health initiatives, research partnerships and policy making, emphasising equitable sharing of resources, knowledge and decision-making power to address health inequities and promote collective solutions to global health challenges. This BMJ Global Health collection highlights the evolution of inclusive internationalism over the past 50 years through the lens of TDR, sponsored by the UN Children’s Fund, the UN Development Programme, the World Bank and the WHO.^6^

The KEP provides a model for how research and innovation directly informed the implementation of a subregional disease elimination programme. It is important to investigate the key elements of KEP and extrapolate lessons for VL elimination programme in East Africa, as well as other diseases targeted for elimination.

## What made the KEP a success?

The KEP depended on a concerted public–private partnership and government commitment. The KEP had strong foundations and took a multisectoral approach. First, there was political commitment, as reflected in the formation of a national KEP in each country and the allocation of resources under national operational plans, in particular in Bangladesh.

Second, the programme could avail of essential tools that had been generated through prior targeted research investments: vector control was already possible through indoor residual spraying and insecticide-treated bed nets, while more innovative methods like insecticide wall painting were also being investigated; an available diagnostic algorithm included a point-of-care rapid diagnostic test (rK39); and VL could be treated with a single infusion of liposomal amphotericin B (LAmB), with backup regimens, including LAmB combined with paromomycin or miltefosine.

Third, investments were made into procuring and delivering tools and implementing strategies to strengthen the public health system through training of healthcare workers and supporting patients.

Finally, country-led efforts were supported by international backing; multiple stakeholders were involved at national and international level, including the public and private sectors, working together to develop and deploy appropriate tools for diagnosis, treatment and prevention of VL.^7 8^

## Applying learning from the KEP to East Africa

In 2022, the East Africa region accounted for 73% of the global VL caseload. In 2023, with WHO support, Chad, Eritrea, Ethiopia, Kenya, Somalia, South Sudan, Sudan, Uganda and Djibouti committed to the elimination of VL as a public health problem, with a target of <1% case fatality from primary VL by 2030^5^: an ambitious timeframe, given the challenges ahead.

East African countries need to thoroughly investigate each element of the strategies that worked in South Asia to identify similarities while also identifying what differs in their local context that requires distinct tools and approaches. A major lesson from the KEP has been that, while having effective tools can trigger an elimination programme, political decisions shape it.

The tools used in the KEP may not be adaptable to the East African context, so development of the tools is needed. Existing diagnostics and treatments—such as the rK39 lateral-flow test and single-dose LAmB—do not perform as well as in South Asia. International research and development collaborations require clear target product profiles informed by the ultimate users in the region, as well as investments that have now significantly slowed down as governments’ and donors’ attention has been diverted to other priorities. Although the global, non-profit research and development organisation ‘the Drugs for Neglected Diseases initiative (DNDi)’ is still pursuing drug development efforts for VL,^9^ no leads are emerging from the diagnostics pipeline, and there are no current prospects for a vaccine. Similarly, insecticide-treated bed nets and insecticide wall paint, which were successful in South Asia in reducing indoor transmission, are not adapted to East Africa’s outdoor transmission.

As in the KEP, East African countries will also need to act on the political commitment of the 2023 Nairobi Declaration. First, by bringing lasting solutions to the several conflicts and the domestic and cross-border migration that are frequent causes of VL outbreaks in the region.

Second, by investing in their healthcare systems and patient support. Reaching VL patients in the region is a challenge: at-risk populations concentrate in difficult-to-access border areas and are mostly displaced populations and migrant workers, who have inadequate access to healthcare and poor knowledge of the disease. Health facilities in these remote places are insufficiently equipped and staffed. Resources need to be mobilised, including domestic and international investments into public health system strengthening to improve access to care, making diagnostics and treatments available, establishing referral systems and training health providers.

Third, countries should lead and shape collaboration with international organisations, such as WHO headquarters and its Regional Office for Africa, TDR, DNDi and the medical humanitarian charity Médecins Sans Frontières to direct resources, research, innovation and implementation. External funds will be essential, especially in the demanding initial high-investment phase of the elimination programme, but governments also need to commit local resources to avoid having to resort to unsustainable external funding or compromising other public health programmes. These efforts must be planned and maintained for as long as needed because decision makers’ and donors’ fatigue can undermine efforts and frustrate future initiatives.

The KEP has shown that endemic countries need to lead the programme. In Bangladesh, a critical success factor of the KEP was its funding, largely from the country’s own resources and mainly country-led research and implementation. VL is one of the many infectious diseases of poverty that disproportionately affects disadvantaged communities and vulnerable people in low-resource countries with limited capacity and resources to research, purchase and deliver medical interventions. This situation has been feeding a dependency spiral that can and must be broken. ‘Decolonising global health’ has garnered considerable attention in recent years,^10^ though how power imbalances can actually be set right is debatable.^11^ Communities and countries must play a central role in resolving their own priority public health problems; international collaborators should provide the support that affected people need.

Major public health achievements cannot happen without affected countries playing a central role in the planning and execution of public health interventions. This was the main driver behind the approach to collaboration adopted by the KEP. This approach also means finding common ground among different stakeholders. For researchers, it means getting out of their comfort zone; learning about the decision-making process; identifying key factors, actors and influencers and targeting the message to the audience and delivering clear, actionable solutions. Funders and international agencies should listen to local actors and not just follow their own priorities and plans. Politicians should commit to VL elimination and stick to their commitments.

## Limitations and challenges

The KEP had limitations and faced challenges. It took 2 years longer than planned in Bangladesh; elimination has not yet been certified in India, despite the country having maintained the required criteria for 2 consecutive years; and Nepal is facing increased geographical spread.

There are also substantial differences between the three countries. India had a case load nearly 5 times Bangladesh’s and more than 20 times Nepal’s, potentially leaving a sizeable human reservoir even after reaching the elimination target.

Nepal faces the opposite challenge of few, interspersed cases in small populations, where even a couple of cases would prevent reaching the target.^4 12^ Both underestimation of the length of commitment and recognition of widely differing scenarios after elimination are directly relevant to planning in the East African context.

It must be made clear that ‘elimination as a public health problem’ does not equate to eradication. The parasite continues to circulate in the human population in the skin of people with post-kala-azar dermal dermatitis, in pockets of high-prevalence VL and possibly in hidden non-human reservoirs, which could trigger outbreaks if surveillance is not in place to detect changing patterns early. Maintaining elimination for any disease has its own challenges and requires new approaches and investments at a time when attention and resources are more thinly spread among myriad priorities.

## Conclusion

In 2019, the WHO heralded the 2020s as ‘the decade for disease elimination’.^13 14^ Halfway through, results have been mixed: eight neglected tropical diseases have been eliminated in at least one country, one of which is VL in Bangladesh.^15^ Cuts to global health funding make future prospects less promising. Therefore now, even more than before, the agenda to eliminate neglected tropical disease needs clear, realistic goals^16^ and a practical roadmap to make it work, which, according to Hietanen *et al* should include eight essential components: country ownership, sustained commitment, dedicated and cross-cutting strategies, integration, one health, stakeholder support, health diplomacy and learning from the past mistakes.^15^ The KEP incorporated many of these elements.

The KEP led to the first declaration of VL elimination in Bangladesh and greatly reduced national, regional and global levels of the disease. The KEP gave us many positive lessons about how local, regional and international teams can work together, with a focus on country leadership, to tackle a complex disease.

While this may give cause for optimism, no single, simple solution can replicate this success in current hotspots, such as East Africa. Elimination of VL in new regions will require local contextualisation of solutions, including new research on disease transmission and innovative outdoor vector control strategies in these geographical settings, and new research on, and development of, adapted diagnostics and therapeutics. Elimination of VL will also require investment for health system strengthening, patient support and improving access to care. Importantly, political commitment needs to include cross-border collaboration. Finally, we must plan carefully for success: KEP has shown very different endpoints and risks of resurgence in different populations that must be tackled in the ongoing maintenance of elimination.

## Data Availability

All data relevant to the study are included in the article.

## References

[1] Steverding D (2017). The history of leishmaniasis. Parasit Vectors.

[2] World Health Organization (2005). Regional strategic framework for elimination of Kala Azar from the South-East Asia Region (2005-2015).

[3] Nagi N (2024). Bangladesh eliminates visceral leishmaniasis. Lancet Microbe.

[4] World Health Organization (2024). Global leishmaniasis surveillance updates 2023: 3 years of the NTD road map. (weekly epidemiological record). report no.: no 45. http://www.who.int/wer.

[5] World Health Organization (2024). Strategic framework for the elimination of visceral leishmaniasis as a public health problem in Eastern Africa 2023–2030.

[6] Reeder J, Aslanyan G, Kitamura M (2024). TDR at 50: advancing a longstanding commitment to inclusion. BMJ.

[7] Special programme for research and training in tropical diseases (2010). Research to support the elimination of visceral leishmaniasis. report no.: TDR/BL10.10.

[8] Hirve S, Kroeger A, Matlashewski G (2017). Towards elimination of visceral leishmaniasis in the Indian subcontinent-Translating research to practice to public health. PLoS Negl Trop Dis.

[9] (2020). Our R&D portfolio for visceral leishmaniasis. https://dndi.org/diseases/visceral-leishmaniasis/projects-achievements/.

[10] Khan M, Abimbola S, Aloudat T (2021). Decolonising global health in 2021: a roadmap to move from rhetoric to reform. BMJ Glob Health.

[11] Chaudhuri MM, Mkumba L, Raveendran Y (2021). Decolonising global health: beyond “reformative” roadmaps and towards decolonial thought. BMJ Glob Health.

[12] Joshi AB, Banjara MR, Chuke S (2023). Assessment of the impact of implementation research on the Visceral Leishmaniasis (VL) elimination efforts in Nepal. PLoS Negl Trop Dis.

[13] (2025). Welcome to 2020 – the decade for disease elimination. https://www.who.int/news-room/feature-stories/detail/welcome-to-2020-the-decade-for-disease-elimination.

[14] Malecela MN, Ducker C (2021). A road map for neglected tropical diseases 2021-2030. Trans R Soc Trop Med Hyg.

[15] Hietanen H, Pfavayi LT, Mutapi F (2025). Unlocking the blueprint to eliminating neglected tropical diseases: A review of efforts in 50 countries that have eliminated at least 1 NTD. PLoS Negl Trop Dis.

[16] Khawar L, Donovan B, Peeling RW (2023). Elimination and eradication goals for communicable diseases: a systematic review. Bull World Health Organ.

